# Editorial: Plant Microbiome: Interactions, Mechanisms of Action, and Applications, Volume II

**DOI:** 10.3389/fmicb.2022.915684

**Published:** 2022-05-31

**Authors:** Alok Kumar Srivastava, Prem Lal Kashyap, Gustavo Santoyo, George Newcombe

**Affiliations:** ^1^National Bureau of Agriculturally Important Microorganisms, Indian Council of Agricultural Research (ICAR), Mau, India; ^2^Indian Institute of Wheat and Barley Research, Indian Council of Agricultural Research (ICAR), Karnal, India; ^3^Instituto de Investigaciones Químico-Biológicas, Universidad Michoacana de San Nicolás de Hidalgo, Morelia, Mexico; ^4^Department of Forest, Rangeland and Fire Sciences, University of Idaho, Moscow, ID, United States

**Keywords:** microbiome, symbionts, biostimulants, metabolites, arbuscular mycorrhizal fungi (AM fungi), rhizosphere, root-knot nematodes, abiotic stress

In a recent article, Suzanne Simard (Simard, 2022) writes of trees that they are “in constant communication with one another through an underground biological neural network made of mycorrhizal fungi.” Networked plants also potentially link other symbionts. Since plants comprise 82% of all living biomass on Earth (Bar-On et al., 2018), their microbiome-enabled network has global reach ecologically. Plant symbionts may mostly be bacteria or fungi but animals (e.g., nematodes), protists, viruses, and even archaeans (Müller et al., 2015) can also integrate into the community. Moreover, when we consider that humanity must depend on this microbially diverse, plant-centered network for food, fiber, optimized carbon sequestration, and conservation of known and unknown biodiversity, it is not difficult to make the case for its importance. Even though we may not yet have seen all the moving parts in action, so to speak, our current studies of facilitated and direct interactions steadily add to our understanding. From a current focus on single plant species, particularly their rhizospheres, we may eventually move to a broad understanding of the collective microbiome of the global plant community.

So, here in Vol. II of the “Plant Microbiome: Interactions, Mechanisms of Action, and Applications” we add 19 articles that collectively address some of the key questions that we can pose and answer today. A total of 15 of the 19 represent reports on original research. The remaining four represent original reviews. The original research articles focus primarily on a range of agricultural crop plants with their associated microbes: cassava, sugarcane, tea, soybean, rice, cotton, Chinese chives, licorice, and tomato. However, also included among the research articles are reports on the effects of symbionts on *Lolium arundinaceum* (tall fescue), *Oxytropis glacialis* (a locoweed species of the Tibetan plateau), and *Ulva fasciata* (a marine green alga). Another research article addresses the interactions of nematophagous fungi and root-knot nematodes, and how those interaction outcomes affect plant health (Kassam et al.).

The four original reviews in this new volume are diverse. In the first, Fadiji et al. provide evidence for specific microbes that can help to mitigate the abiotic stresses of climate change. These microbial biostimulants might be called adaptogenic, since they can help plants to adapt to extreme weather events that are becoming more common with continuing climate change. Although the COVID pandemic temporarily reduced anthropogenic emissions of greenhouse gasses, deepening concern over the effects of climate change is warranted (Depledge et al., 2022). Excessive heat, untimely frosts, salinity and drought, and flooding threaten the viability of agriculture everywhere. Those microbes singled out for discussion of their protection of crop plants against extreme heat and cold are primarily bacterial taxa, although species of *Glomus*-forming arbuscular mycorrhizae are also included. The same is true for protection against drought and salinity stress, although, *Penicillium* spp. are included with *Glomus* spp. in the fungal category.

The second review by Suman et al. is complementary to that of Fadiji et al.. Microbial bioinoculants are discussed as essential adjuncts to sustainable agriculture. Sustainability is assumed to include an adaptive response to climate change mediated by microbes. Again, bacteria are emphasized in terms of their ability to stimulate growth of their hosts, along with their ability to defend plants. Some fungal examples are also covered in this article review by Fadiji et al..

The third review by Mishra et al. focuses on metabolomic insights into endophyte-derived compounds that affect plant performance. Early in their review, the authors emphasize that although plants themselves produce important secondary metabolites, their endophytes do as well. The list of these dual-source metabolites includes medically important compounds such as taxol, vincristine, vinblastine, camptothecin, and hypericin. Additionally, metabolites in terms of their functions in plant defense have also been discussed.

In another review, Chot and Reddy focus on the growth of ectomycorrhizal (ECM) plants on land contaminated with heavy metals. For many years, phytoremediation has included the effects of symbionts (Newman and Reynolds, 2005). The authors stated that this particular subject (i.e., remediation with ECM plants) is “highly under-studied,” but that the probable mechanisms include the following: (1) physical intervention (presumably of the ECM mantle) such that metals in soil do not contact plant tissue; (2) enhanced antioxidant activity and metal sequestration; and (3) mycorrhiza-altered nutrient uptake that increases tolerance of metals.

With respect to the reports of original research, we turn first to endophytes of *Oxytropis glacialis* (a locoweed species of the Tibetan plateau). Locoweeds are defined as plant species that contain a toxic alkaloid, swainsonine, that can cause locoism in grazing animals; species of both *Astragalus* and *Oxytropis* have been implicated. Braun et al. (2003) were the first to show that endophytes in locoweeds could produce swainsonine. In this volume, Cao et al. reported fungal diversity in the soil of roots of *Oxytropis glacialis* and reported OTUs associated with swainsonine producers.

Arbuscular mycorrhizal fungi (AMF) can affect floral traits, fruit yield, resource allocation, and seed germination of cherry tomato. In this volume, Wang L. et al. showed that nutrient levels can interact with AMF treatments to affect plant responses. It has been highlighted that tall fescue (*Lolium arundinaceum*) is affected by both *Epichloë* endophytes and AMF. *Epichloë* endophytes can aid tall fescue under conditions of saline-alkali stress. The beneficial effect of endophytes under these conditions was improved by one AMF, *Claroideoglomus etunicatum*, but was hindered by a second AMF, *Funneliformis mosseae*. Liu H. et al. thereby highlight the interactions of key symbionts of particular plants.

Cassava (*Manihot esculenta*) is an important staple and cash crop in tropical and subtropical regions, where its tolerance of harsh conditions (drought, in particular) is valued. According to Ha et al., the microbial community of cassava is not adequately studied. Using 14 cassava genotypes, bacterial 16S rDNA sequencing revealed that the richness and diversity of bacteria in the rhizosphere were higher than those in the tuber endosphere. Cassava genotype did affect rhizosphere bacterial communities but not endophytic tuber communities. The bacterial phyla of tubers and rhizosphere were Bacillota (formerly Firmicutes) and Actinomycetota (formerly Actinobacteria), respectively. This study should pave the way for further exploration of the cassava microbiome.

Two sugarcane varieties with differing drought tolerance were the basis for an interesting study by Liu Q. et al. of the function of rhizosphere bacteria in response to water stress. One variety was tolerant; the other was sensitive. Rhizosphere bacterial diversity was reduced by drought unequally in the two varieties: more in the sensitive than in the tolerant variety. Drought-resistant bacteria persist in the rhizosphere of the tolerant variety but do not in that of the sensitive one. This interesting paper should prompt confirmatory studies in additional plant systems.

Positive effects on plant growth and development are associated in particular with these bacterial genera: *Arthrobacter, Azoarcus, Azospirillum, Azotobacter, Bacillus, Burkholderia, Curtobacterium, Erwinia, Gluconobacter, Klebsiella, Serratia, Pantoea, Herbaspirillum, Rahnella, Pseudomonas*, and *Xanthomonas* (Compant et al., 2011; Kushwaha et al., 2020; Hazarika et al.). The latter authors tackled the culturable, endophytic community of bacteria in leaves and roots of tea (*Camellia sinensis*) in northeastern India. They then focused on *in vitro* and *in planta* traits for plant growth promotion. A total of 106 isolates were identified for further development as bioinoculants that would greatly aid sustainable tea production.

Yuan et al. investigated the expression of *Bradyrhizobium diazoefficiens* genes in nodule samples from five developmental stages of soybean (i.e., branching, flowering, fruiting, pod maturation, and harvest). Their findings hint at further improvements in engineering efficiency of nitrogen fixation.

Chu et al. found that six bacterial phyla characterized the microbiome of roots of six rice cultivars grown in cadmium-contaminated paddy fields in China: Pseudomonadota (formerly Proteobacteria), Bacillota (Firmicutes), Actinomycetota (Actinobacteria), Acidobacteriota (Acidobacteria), Bacteroidota (Bacteroidetes), and Spirochaetota (Spirochaetes). Their most interesting finding is that these endophytes form “a more highly interconnected network and exhibit higher operational taxonomic unit (OTU) numbers, diversities, and abundance” during the reproductive phase of their rice host than during the vegetative stage. Some of the root endophytes also displayed resistance to cadmium, which points to possible applications.

Xinjiang is an arid region of China in which cotton is grown. Shi et al. compared “the rhizosphere and endosphere microbiomes of healthy and diseased cotton from north and south of the Tianshan Mountains using the methods of PCR-based high-throughput sequencing and real-time quantitative PCR.” Interestingly, diseased plants hosted a greater abundance of both rhizosphere and endosphere bacteria and fungi.

Persistent application of chemical fertilizers to sugarcane crops can lead to pollution, degradation of the soil biota, and yield losses in the long term (Robinson et al., 2011). In an ambitious project reported in this volume (Singh et al.), endophytic root bacteria were screened for their potential as biofertilizers. The following two isolates were most promising: *Pantoea cypripedii* AF1 and *Kosakonia arachidis* EF1. Both strains readily colonized the leading Chinese sugarcane.

A popular, perennial vegetable in China (i.e., Chinese chives, or *Allium tuberosum*) was the focus of a study by Sun et al. of the plant's endophytic bacteria. Bacteria with high relative abundance in an ecological compartment acted as key nodes.

Microbial symbionts of green algae also make an impressive appearance in this volume (Wang H. et al.). Among epiphytic bacteria, a new species, *Hyunsoonleella* sp. HU1-3 (family Flavobacteriaceae), was identified which significantly promoted the growth of its green algal host, *Ulva fasciata*.

Root-knot nematodes are the target of a study (Kassam et al.) employing Indian isolates of two nematophagous fungi: *Arthrobotrys thaumasia* and *Tolypocladium cylindrosporum*. These fungi show potential as biocontrol agents of the plant-parasitic nematodes that are responsible for significant crop damage globally.

From the sugarcane rhizosphere, 58 actinobacteria were isolated and evaluated for their antagonistic potential in a study by Wang Z. et al.. Smut resistance was the focus, and *Streptomyces griseorubiginosus* showed considerable potential as it possessed the metabolic tools to alleviate biotic stress factors like smut fungi.

Wild licorice (*Glycyrrhiza uralensis*) has been an important traditional medicine in China. With wild populations dwindling, best practices for cultivation are now being researched (Yue et al.). Drought is a significant abiotic stress factor for licorice. In prior research, *Bacillus amyloliquefaciens* FZB42 was shown to confer stress tolerance to *Arabidopsis*. Here, the authors demonstrated that this strain could alleviate drought stress; it also increased yields of total flavonoids, liquiritin, and glycyrrhizic acid which are the medicinal products of *G. uralensis*.

In conclusion, after review of the 19 articles of Vol. II, one thing is clear: the “exciting possibilities” of the plant-microbe interactome, to which we alluded in Vol. I, are now bearing fruit. We can expect many more exciting discoveries in the upcoming years.

## Author Contributions

All authors listed have made a substantial, direct, and intellectual contribution to the work and approved it for publication.

## Conflict of Interest

The authors declare that the research was conducted in the absence of any commercial or financial relationships that could be construed as a potential conflict of interest.

## Publisher's Note

All claims expressed in this article are solely those of the authors and do not necessarily represent those of their affiliated organizations, or those of the publisher, the editors and the reviewers. Any product that may be evaluated in this article, or claim that may be made by its manufacturer, is not guaranteed or endorsed by the publisher.
